# A Cross-Sectional Survey Exploring the Impact of the COVID-19 Pandemic on the Cancer Care of Adolescents and Young Adults

**DOI:** 10.3390/curroncol28040278

**Published:** 2021-08-22

**Authors:** Kaitlyn Howden, Camille Glidden, Razvan G. Romanescu, Andrew Hatala, Ian Scott, Julie Deleemans, Karine Chalifour, Geoff Eaton, Abha A. Gupta, James M. Bolton, Alyson L. Mahar, Sheila N. Garland, Sapna Oberoi

**Affiliations:** 1 Department of Pediatrics and Child Health, University of Manitoba, Winnipeg, MB R3A1S1, Canada; khowden2@manitoba-physicians.ca; 2 Department of Psychiatry, University of Manitoba, Winnipeg, MB R3E3N4, Canada; cglidden@manitoba-physicians.ca (C.G.); JBOLTON@hsc.mb.ca (J.M.B.); 3 George & Fay Yee Centre for Healthcare Innovation, University of Manitoba, Winnipeg, MB R3E0T6, Canada; Razvan.Romanescu@umanitoba.ca; 4 Department of Community Health Sciences, Max Rady College of Medicine, University of Manitoba, Winnipeg, MB R3E0W3, Canada; Andrew.Hatala@umanitoba.ca (A.H.); Alyson.Mahar@umanitoba.ca (A.L.M.); 5 Department of Psychosocial Oncology, CancerCare Manitoba, Winnipeg, MB R3E0V9, Canada; iscott@cancercare.mb.ca; 6 Department of Psychosocial Oncology, University of Calgary Cumming School of Medicine, Calgary, AB T2N4N2, Canada; julie.deleemans@ucalgary.ca; 7 Young Adult Cancer Canada, St. John’s, NL A1A5B5, Canada; karine@youngadultcancer.ca (K.C.); geoff@youngadultcancer.ca (G.E.); 8 Princess Margaret Cancer Care Research Institute, Toronto, ON M5G2C1, Canada; abha.gupta@sickkids.ca; 9 Department of Pediatric Hematology-Oncology, The Hospital for Sick Children, Toronto, ON M5G1X8, Canada; 10 Manitoba Centre for Health Policy, University of Manitoba, Winnipeg, MB R3E3P5, Canada; 11 Research Institute in Oncology and Hematology, CancerCare Manitoba, Winnipeg, MB R3E0V9, Canada; 12 Department of Psychology, Memorial University, St. John’s, NL A1C5S7, Canada; sheila.garland@mun.ca; 13 Department of Pediatric Hematology-Oncology, CancerCare Manitoba, Winnipeg, MB R3E0V9, Canada; 14 CancerCare Manitoba Research Institute, CancerCare Manitoba, Winnipeg, MB R3E0V9, Canada

**Keywords:** COVID-19, adolescents and young adults, cancer, oncology, virtual care, pandemic

## Abstract

We aimed to describe the negative and positive impacts of changes in cancer care delivery due to COVID-19 pandemic for adolescents and young adults (AYAs) in Canada, as well as the correlates of negative impact and their perspectives on optimization of cancer care. We conducted an online, self-administered survey of AYAs with cancer living in Canada between January and February 2021. Multiple logistic regression was used to identify factors associated with a negative impact on cancer care. Of the 805 participants, 173 (21.5%) experienced a negative impact on their cancer care including delays in diagnostic tests (11.9%), cancer treatment (11.4%), and appointments (11.1%). A prior diagnosis of mental or chronic physical health condition, an annual income of <20,000 CAD, ongoing cancer treatment, and province of residence were independently associated with a negative cancer care impact (*p*-value < 0.05). The majority (*n* = 767, 95.2%) stated a positive impact of the changes to cancer care delivery, including the implementation of virtual healthcare visits (*n* = 601, 74.6%). Pandemic-related changes in cancer care delivery have unfavorably and favorably influenced AYAs with cancer. Interventions to support AYAs who are more vulnerable to the adverse effects of the pandemic, and the thoughtful integration of virtual care into cancer care delivery models is essential.

## 1. Introduction

COVID-19 is responsible for one of the largest global pandemics experienced in almost a century [1]. Individuals of all ages with a cancer diagnosis are particularly vulnerable to experiencing a multitude of adverse impacts secondary to the pandemic [2,3]. The enormous strain of COVID-19 on healthcare systems has significantly disrupted cancer care services worldwide, resulting in delayed diagnosis and treatment of patients with cancer and a reduction in their chances of survival [3,4,5,6]. Individuals with cancer are more prone to complications related to COVID-19 infection due to the underlying immune suppression and co-morbidities resulting from cancer and its treatment [7]. Guidelines on how to navigate the provision of healthcare during this pandemic have been rapidly changing and region specific, making it a challenging time for healthcare providers providing cancer care [8].

To combat the spread of the COVID-19 virus, most cancer care organizations have decreased the frequency of in-person appointments and diagnostic tests and shifted towards the provision of healthcare services through virtual platforms [3,4,5,6]. Alterations in cancer treatment protocols and access to clinical trials, medications, and physical or psychological support services have also raised concerns about the quality of cancer care during this pandemic [6,8,9]. Furthermore, it has become more challenging for patients to cope with their cancer given the personal disconnect they feel from healthcare providers and their social support system [5,6].

Adolescents and young adults (AYAs) diagnosed with cancer between the ages of 15 and 39 years have felt the impact of shifting cancer care dynamics; their unique developmental, educational, social, and emotional needs may put them at a higher risk of experiencing adverse consequences of this pandemic [10]. A recently conducted online survey of 177 AYAs with cancer found that 45% of AYAs had their cancer care impacted by this pandemic, including delays in appointments and treatment and alterations to treatment protocols [11]. This survey included only 93 AYAs from North America and was conducted during an early phase of the pandemic. Furthermore, it did not identify the subgroups of AYAs that are more vulnerable to the negative cancer care impact or examine the perceived positive effects of changes to cancer care delivery during pandemic. Given the prolonged duration of this pandemic and the dynamic nature of public health restrictions, it is crucial to understand the cumulative effect of this pandemic on the cancer care of this population. Such data can help to address the current and future needs of AYAs with cancer.

Therefore, we sought to study the impact of the COVID-19 pandemic on AYAs with cancer (ICOVIDAYA) in Canada. Our analysis aimed to describe both the positive and negative impacts of the COVID-19 pandemic-related changes in cancer care delivery on AYAs with cancer, to identify the sociodemographic-, cancer-, and health-related factors associated with negative impacts, and to identify AYAs’ perspectives on the optimization of cancer care during and after the pandemic.

## 2. Methods

We conducted a national, cross-sectional, self-administered online survey of AYAs living in Canada. All AYAs ≥ 18 years old diagnosed with cancer between the ages 15 and 39 years and living in Canada at the time of survey completion were eligible for this study. For this particular analysis, we included the AYAs who were between 18 and 39 years of age at the time of completion of the survey. Questions of our survey were built upon a prior survey used for the Young Adults with Cancer in their Prime (YACPRIME) study and by reviewing the current literature [11,12]. The survey questionnaire underwent multiple iterations through virtual meetings with co-investigators and AYA cancer survivors. The final survey questionnaire contained 49 questions and required 10–15 min to complete. The select questions related to this analysis are listed in Supplementary Appendix A. We translated the final survey instrument into French and then back translated it into English for accuracy; the survey was offered in both official languages to the participants. The questionnaire domains pertaining to this analysis included sociodemographic and cancer-related information and the effects of the COVID-19 pandemic on cancer care. The University of Manitoba’s Research Ethics Board approved this study (HS: 24501).

### 2.1. Survey Administration

This survey was created and administered online using REDCap, which is a secure web application [13]. A convenience sample of AYA participants were recruited across Canada by sharing the online survey link through social media sites of the Young Adult Cancer Canada (YACC) group and other Canadian AYA support groups, as well as through oncology clinics at CancerCare Manitoba. Patient partners also aided in disseminating our survey within their networks [12]. The survey was open for completion between January and February 2021. Informed consent was obtained online and was required before participating in the survey (Supplementary Appendix A). On completion of the survey, participants were offered a CAD 10 e-gift card. Contact details provided for this purpose were not linkable to the participants’ survey responses.

### 2.2. Survey Measures and Definitions

#### 2.2.1. Measuring a Negative and Positive Impact on Cancer Care Delivery

Participants were asked to rate the satisfaction of their cancer care during the pandemic using a 5-point Likert scale from very satisfied to very dissatisfied. A negative impact on cancer care was defined as a participant experiencing any of the following outcomes: changes to the treatment protocol; lack of access to clinical trials; delay or cancellation of appointments, diagnostic testing, or treatment; or limited access to supportive care resources such as mental health, spiritual therapy, physiotherapy, occupational therapy, and vocational rehabilitation. We did not consider changing in-person appointments to virtual appointments (phone-call, video-call, telehealth) as a negative impact. To assess the changes in cancer care delivery associated with a positive impact, participants were asked, “What changes in cancer care delivery during the pandemic have had a positive effect on you?”. For this question, participants could select as many options as relevant from the options of, telephone visits, telehealth visits, videocall visits, delivery of cancer care closer to home, ability to communicate with healthcare providers by phone or email, and “other”. Participants’ perspectives on optimizing cancer care delivery during the pandemic were elicited through a free-text question, “How can cancer organizations provide optimal cancer care during this pandemic to adolescents and young adults with cancer?”.

#### 2.2.2. Measuring Sociodemographic-, Cancer-, and Other Health-Related Information

Sociodemographic data obtained from participants included age, gender (man/woman), province or territory of residence, geographic area (urban/rural/remote), race/ethnicity (white/non-white), relationship status (in a relationship/single), education status (full-time student/part-time student), employment status (employed/unemployed/disability or unemployment benefits), impact of COVID-19 on employment (yes/no), and personal income in the year 2020 in CAD (<20,000/20,000–40,000/40,000–60,000/>60,000). Cancer-related information collected from participants included type of cancer diagnosis (hematologic vs. non-hematological), time since cancer diagnosis (<2 years/2 to <5 years/≥5 years) and presence of ongoing cancer treatment (yes/no). The participants self-reported the presence of a pre-pandemic mental illness such as anxiety, post-traumatic stress, and obsessive-compulsive or mood disorders. Participants stated current diagnoses of chronic physical health conditions, such as hypertension, diabetes, stroke, or other organ dysfunction, and this information was used to identify the presence of a chronic physical health condition (yes/no).

#### 2.2.3. Province or Territory of Residence

Residential information was collapsed into five geographic regions: Central Canada (Ontario, Québec), Prairies (Alberta, Manitoba, Saskatchewan), British Columbia, Atlantic Canada (New Brunswick, Newfoundland and Labrador, Nova Scotia, Prince Edward Island), and Territories (Northwest Territories, Nunavut, Yukon).

### 2.3. Statistical Analysis

We summarized the sociodemographic-, cancer-, and other health-related data using descriptive statistics. Chi-square tests for independence and logistic regression were used to test associations between the pre-determined sociodemographic-, cancer-, and health-related variables and a negative impact on cancer care (yes/no). Odds ratios (OR) with associated 95% confidence intervals (CI) were used to report the associations. Using the Benjamin–Hochberg test for multiple testing, a *p*-value less than 0.03 was considered statistically significant for univariable analysis [14].

All variables were then included in a single multivariable logistic regression model to explore their independent associations with experiencing a negative impact on cancer care, adjusting for all other factors in the model. We excluded participants with one or more missing values for any of the multivariable logistic regression variables. Multicollinearity between dependent variables was assessed using the correlation matrix and variation inflation factor [15]. As a secondary analysis, we also examined the factors independently associated with a negative impact on cancer care for those on active cancer treatment compared to those who had completed cancer treatment using multiple logistic regression. All tests were two-sided, with a *p* < 0.05 indicating statistical significance for multiple logistic regression. All the analyses were done using R software (version 4.0.0) [16].

### 2.4. Qualitative Analysis

Qualitative responses to an open-ended question on optimizing cancer care underwent summative content analysis [17]. We used Dedoose software (version: 8.3.47) for this analysis [18]. The codes were independently generated by KH and CG and were further refined by input from SO and AH. During the initial coding, the data were read thoroughly to determine the underlying meaning being conveyed by respondents, and all possible ideas were created inductively from the data to preserve the participants’ original meaning. We then pursued a set of central codes to all the excerpts during focused coding. The codes were organized into various categories during theoretical coding. The categories were refined throughout the analysis and were compared to one another using the constant comparative method to generate final themes [19].

## 3. Results

### 3.1. Patient Demographics

The survey was completed by 1063 participants, of which 805 were included in the analysis. We excluded 258 participants either because of age >39 years (*n* = 138) or not reporting their age (*n* = 120). Missing data were minimal (0.1–2%). Table 1 summarizes the demographic and clinical characteristics of the study population. The mean age of participants was 30.3 years (SD = 5.3); 172 (21.4%) were between 18 and 25 years of age. Most participants identified themselves as white (*n* = 770, 95.6%) and lived in urban areas (*n* = 605, 75.5%). The most common cancer diagnosis was a solid tumor (non-brain tumors) (*n* = 615, 76.4%). Most participants were less than five years from the time of cancer diagnosis (*n* = 667, 82.8%), and one-third were receiving some form of cancer treatment (*n* = 265, 33.0%). Pre-pandemic mental illness and chronic physical illness were reported by 17.8% (*n* = 118) and 23.9% (*n* = 192) of participants, respectively. Of the 805 included survey responses, 235 open-ended responses were provided totaling 2072 words.

### 3.2. Impact of COVID-19 Pandemic on Cancer Care

**Figure 1:**
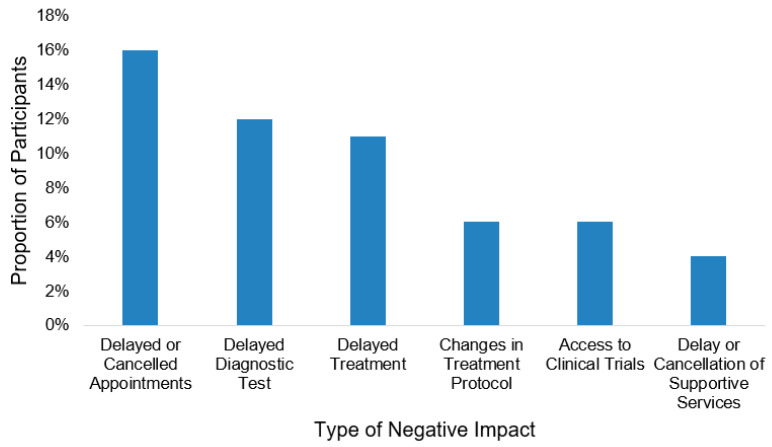
Type of negative impact of the COVID-19 pandemic on cancer care (*n* = 173).

**Figure 2:**
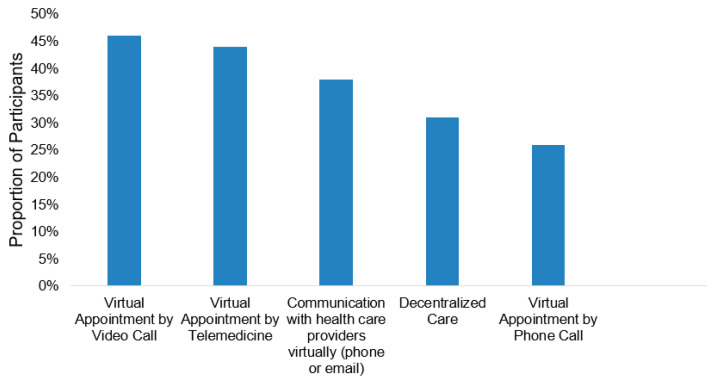
Changes in cancer care delivery associated with a positive impact (*n* = 767).

Overall, 76.6% reported being satisfied with their cancer care during the pandemic (*n* = 617). In total, 173 participants (21.5%) experienced a negative impact on their cancer care due to the pandemic. Among those receiving active cancer treatment (*n* = 265), 27.2% (*n* = 72) encountered a negative impact on their cancer care. Overall, the most common negative impacts were delays or cancellations in either appointments (*n* = 131, 16.2%) diagnostic testing (*n* = 96, 11.9%), or treatment (*n* = 92, 11.4%) (Figure 1). Of those experiencing a negative impact, 156 (19.4%), 103 (12.8%), and 49 (6.1%) experienced at least one, two, and three of these impacts, respectively. Almost all participants experienced positive impacts due to pandemic-related changes to the delivery of their cancer care (*n* = 767, 95.2%). Three-quarters of the participants (*n* = 601, 74.6%) responded that virtual visits had a positive impact on them, particularly the video call visits (*n* = 357, 44.3%) and telehealth visits (*n* = 368, 45.7%) (Figure 2). Receiving cancer care closer to home (*n* = 217, 28.6%) and the ability to communicate with healthcare providers outside of appointments via phone calls (*n* = 217, 27.0%) and emails (*n* = 98, 12.2%) also positively influenced cancer care (Figure 2).

### 3.3. Factors Associated with a Negative Impact of COVID-19 Pandemic on Cancer Care

On univariable analyses, woman gender, province/territory of residence, pre-pandemic mental illness or chronic physical health condition, and ongoing cancer treatment were associated with experiencing at least one negative impact on cancer care (*p* < 0.03). After excluding 98 individuals due to variable selection, 707 participants were included in the multivariable regression. A pre-existing mental illness (OR = 12.14, 95% CI 6.98–21.66, *p* < 0.001), having a chronic physical health condition (OR = 2.22, 95% CI 1.34–3.67, *p* = 0.002), personal income <20,000 CAD in the year 2020 (OR = 4.21, 95% CI 2.03–8.75), and ongoing cancer treatment (OR = 1.69, 95% CI 1.03–2.77, *p* = 0.036) were independently associated with a negative impact on cancer care (Table 2). The odds of having a negative impact on cancer care were also higher among participants from Central Canada (OR = 8.43, 95% CI 2.90–32.03, *p* < 0.001), the Prairies (OR = 4.52, 95% CI 1.54–17.13, *p* = 0.012), and British Columbia (OR = 6.74, 95% CI 2.05–27.63, *p* = 0.0034) when compared to those living in the Territories of Canada.

When comparing the multivariable regression analyses examining the independent factors associated with a negative impact on cancer care for those on active cancer treatment to those off treatment, the presence of a pre-existing mental illness, Province/Territory of residence, and a personal income <20,000 CAD in the year 2020 remained statistically significant for both groups (Tables S1 and S2). While the presence of a pre-pandemic chronic physical health condition was significant for those on active cancer treatment (OR = 6.88, 95% CI 2.18–22.14, *p* = 0.001), it was not statistically significant for those who had completed treatment (OR = 1.40, 95% CI 0.75–2.57, *p* = 0.281). Significance of gender also differed between the two groups; being a woman was independently associated with a negative impact on cancer care for those belonging to the completed treatment group (OR = 2.22, 95% CI 1.30–3.82, *p* = 0.004) but not for those in the active treatment group (OR = 0.76, 95% CI 0.28–1.94, *p* = 0.567).

### 3.4. Optimization of Cancer Care

Table 3 contains the common themes and subthemes and relevant excerpts that emerged from participants’ responses (*n* = 235, 29.2%) on how cancer care could be optimized during the pandemic. The main themes included improving healthcare visits, enhancing personalized care, improving prevention of COVID-19, addressing information needs, and avoiding diagnostic and treatment delays. The majority wanted the continuation of virtual care, including increased opportunities to connect with their healthcare team via emails and phone calls.

*“Provide telephone and network consultation services to reduce the number of visits to the hospital and increase the function of making an appointment in advance.”*

Many patients reported they preferred virtual options with a video component as it helped them feel more connected to their healthcare provider and reduced their fear of missing their health issues compared to phone visits.

*“Need more access electronically to supports (texts, email, etc.), would like access to test results, I think video chat is better than phone calls.”*

Whereas some participants wanted the ability to choose between in-person and virtual appointments and favored in-person visits over virtual visits:

*“I think telephone and virtual appointments are only helpful for some people and should not be a catchall. I would prefer to see more in-person options for those that are struggling.”*

The participants also expressed a desire for their healthcare team to provide more educational resources related to their cancer care, self-care, and navigating the COVID-19 pandemic as a patient with cancer. Some participants highlighted that their concerns were not being listened to or addressed and that their healthcare teams must improve communication.

## 4. Discussion

Our cross-sectional study highlighted both negative and positive aspects of how the COVID-19 pandemic modified cancer care delivery to AYAs with cancer in Canada. To our knowledge, this is the first Canadian study to assess in detail how the pandemic has impacted multiple aspects of cancer care of AYAs with cancer and to identify factors that place certain AYAs with cancer at greater risk for experiencing the negative impact of this pandemic.

We found that the pandemic unfavorably altered the cancer care of one-fifth of AYAs diagnosed with cancer. Notably, other studies have reported higher rates (30–50%) of the negative impact of the pandemic on cancer care [20,21,22]. These differential findings are likely attributable to the lower number of patients receiving active cancer treatment in our study compared to other studies and the different geographic locations, waves of the pandemic, and healthcare systems between the studies [11,20,21,22].

In our study, the most frequently experienced negative impacts on care were delays or cancellations in diagnostic tests, treatment, or healthcare visits. These findings are congruent with other studies demonstrating a similar spectrum of pandemic-related delays in diagnosis and staging, initiation of therapy, and interruption of ongoing treatment and clinical research [3,4,5,6]. AYAs with pre-existing mental health or chronic physical health conditions were at the highest risk of having a negative impact. AYAs with comorbid physical and mental health conditions may worry more about acquiring the COVID-19 infection than others and might be more hesitant to visit hospitals [11]. The COVID-19 pandemic has further increased the levels of distress for those with pre-existing mental health conditions by diminishing access to mental health support and the typical coping strategies such as family and friend support systems [23,24]. Those with chronic health conditions and limited support may face increased difficulties accessing and navigating their cancer care during the pandemic [25]. Those in the active phase of treatment typically require more frequent visits to the hospital than those who are off treatment, and this may explain why a chronic health condition had more of a negative impact on cancer care for this subgroup of AYAs.

We found that participants in the lowest income bracket (<20,000 CAD) were also at an elevated risk of encountering an adverse impact on their cancer care compared to others, even in Canada’s publicly insured healthcare system. Pre-pandemic studies have shown disparities in cancer care for socioeconomically disadvantaged individuals [26]. These individuals have also been disproportionally affected by this pandemic in different ways, including having a higher likelihood of being employed in jobs that do not provide the option of working from home or of paid leaves [27]. Access to cancer care may also be challenging for this subgroup of patients because of not having the safe and reliable means to travel to appointments or the inability to access virtual platforms used for cancer care delivery during pandemic [24]. We also observed different experiences of negative impacts on cancer care across the provinces and territories in Canada. The substantial variability in COVID-19 infection and mortality and public health restrictions across Canada may have influenced cancer care delivery differently across the country [27].

When comparing factors that had a negative impact on cancer care between those actively receiving treatment and those off therapy, identifying as a woman was only significant for the off-therapy group. One possible explanation for this is that those who have completed treatment were often considered better candidates to receive part of their ongoing follow-up care through virtual care during this pandemic. While most studies have found that gender does not play a significant role in determining satisfaction with telemedicine appointments, one recent retrospective cohort study looking at appointments conducted during the pandemic found that women experienced lower patient satisfaction during virtual visits as compared to men [28,29,30,31,32]. The lower satisfaction level with virtual appointments during the survivorship period might have been perceived as having a negative impact on their overall cancer care by women.

Despite the disruptions in cancer care, most AYAs reported positive effects resulting from the virtual care and increased decentralization of cancer care during the pandemic. In particular, virtual care with a video element was favored by most. Virtual healthcare has many benefits, including increasing safety for healthcare providers and patients by allowing for connectivity from a distance and increasing the accessibility for those who struggle to attend in-person appointments due to location or time constraints [33]. While the concept of virtual care is not new in oncology, the widespread delivery of virtual care increased significantly during pandemic to protect patients from COVID-19 infection [34]. Although most participants in our study wanted to include some form of video calls or telehealth into their cancer care, this was not a desirable option for all. Integrating virtual care into routine cancer care must be based on each patient’s values and preferences and should be the focus of future research; it should not increase the inequalities in cancer care due to the existing digital divide.

Some limitations of our study deserve attention. Most participants completing the survey were white, identified as men or women, and were from urban areas. As a result, our survey does not capture cancer care delivery experiences of Black or Indigenous AYAs, AYAs of color, gender diverse AYAs, and those living in remote geographical locations. More research and outreach are needed to traditionally underserved AYA cancer populations to understand their care experiences during the pandemic and ensure their perspectives are included in optimizing cancer care moving forward. Since we only surveyed AYAs with cancer in Canada, our findings may not be generalizable to those living in other countries with different healthcare systems and varying pandemic severity. Although not classified as having a negative impact, virtual care might have unfavorably affected some AYAs, as was apparent from the qualitative data. Our survey also might have inflated the positive influence of virtual care by selecting individuals with access to the technology required for completing this online survey. Finally, ascertainment of pre-existing mental and physical health conditions was based on self-reporting and is subject to social desirability bias [35].

Despite these limitations, our study has several strengths. Our study captured the impact of the pandemic on cancer care of AYAs with cancer who have unique needs and experiences compared to other cancer populations. A large and diverse sample size of AYAs with various cancer diagnoses from different geographical locations across the province participated in our survey. Besides collecting quantitative data, we also received diverse qualitative responses from study participants. The qualitative responses provided more depth and richness to our understanding of the pandemic’s impact and corroborated our quantitative findings [36]. Insights gained from these patient experiences can provide further guidance on the strategies needed to improve the quality of life and healthcare delivery to AYAs with cancer throughout the remainder of the pandemic and into the post-pandemic era.

In conclusion, despite the remarkable feats of adaptation by cancer organizations to provide essential care during this pandemic, one in five AYAs in Canada experienced a negative impact on their cancer care delivery. Those with pre-existing mental and chronic physical health diagnoses require additional strategies to navigate cancer care during the pandemic. Greater advocacy and supports are essential for socioeconomically disadvantaged individuals to assure equitable access to cancer care. Thoughtful integration of virtual care into ambulatory care can improve cancer care delivery to AYAs even in the post-pandemic landscape.

## Figures and Tables

**Table 1:** Descriptive characteristics of the study population (*n* = 805).

Variable	Mean ± SD, or *n*	% (Range)
Age (in years)	30.27 ± 5.27	(18–39)
Gender ^a^		
- Man	445	55.50%
- Woman	357	44.50%
- Non-Binary	3	0.00%
Ethnicity (White)	770	95.60%
Relationship Status (in a relationship)	484	60.10%
Province/Territory		
- Prairies ^e^	233	28.90%
- Central Canada ^f^	222	27.60%
- Atlantic ^g^	169	21.00%
- Territories ^h^	93	11.60%
- British Columbia	88	10.90%
Geographic Location ^b^		
- Urban	605	75.50%
- Rural	179	22.30%
- Remote	17	2.10%
Education Status ^c^		
- Part-time student	24	3.00%
- Full-time student	75	9.30%
Employment Status ^c^		
- Employed (part or full time)	562	70.00%
- Unemployed	103	12.80%
- Disability or unemployment benefits	32	4.00%
- Other ^i^	51	6.30%
Personal Income in year 2020 ^j^		
- <$20,000	57	7.10%
- $20,000 to <$40,000	115	14.30%
- $40,000 to <$60,000	195	24.30%
- ≥$60,000	389	48.40%
Pre-pandemic mental health condition (yes) ^d^	118	14.80%
Type of pre-pandemic mental health condition ^d^		
- Anxiety disorder	75	9.40%
- Mood disorder	65	8.10%
- Other ^k^	4	0.50%
Presence of a chronic physical health condition (yes)	192	23.90%
Type of chronic physical condition		
- Hypertension or Diabetes	89	11.00%
- Lung or Heart Disease	59	7.30%
- Kidney or Liver Disease	58	7.20%
- Other ^l^	22	2.70%
Cancer type		
- Hematological malignancies	155	19.30%
- Solid tumors (non-brain tumors)	615	76.40%
- Brain tumors	35	4.30%
Time since cancer diagnosis		
- <2 years	246	30.50%
- 2 years to <5 years	421	52.20%
- ≥5 years	138	17.10%
Currently receiving cancer treatment (yes) ^c^	265	33.00%
Negative impact of COVID-19 pandemic on cancer care		
- Yes	173	21.50%
- No	632	78.50%

^a^ *n* = 802; ^b^ *n* = 801; ^c^ *n* = 803; ^d^ *n* = 800, ^e^ Alberta, Manitoba, Saskatchewan; ^f^ Ontario, Quebec; ^g^ Newfoundland and Labrador, Nova Scotia, New Brunswick, Prince Edward Island; ^h^ Yukon, Northwest Territories, Nunavut; ^i^ e.g., Caregiver/Homemaker; Leave of Absence; ^j^ in Canadian Dollars; ^k^ e.g., Personality disorder, ADHD; ^l^ e.g., Stroke, autoimmune diseases, seizure disorder.

**Table 2:** Factors associated with a negative impact of the COVID-19 pandemic on cancer care delivery.

	Univariable Analysis (*n* = 805)			Multivariable Analysis (*n* = 707)		
	Adjusted Odds Ratio	95% CI (Lower-Upper)	*p*-Value	Adjusted Odds Ratio	95% CI (Lower, Upper)	*p*-Value
Age						
- >25 years	0.72	0.49–1.07	0.102	0.98	0.94–1.03	0.431
- <18–25 years	(ref)			(ref)		
Gender ^a^						
- Woman	1.73	1.23–2.43	0.001	1.41	0.90–2.20	0.13
- Man	(ref)			(ref)		
Ethnicity						
- Non-White	1.8	0.83–3.89	0.133	2.07	0.76–5.52	0.147
- White	(ref)			(ref)		
Province/Territory						
- Central Canada ^e^	9.41	3.32–26.69		8.43	2.90–32.03	<0.001
- Prairies ^f^	7.37	2.59–20.96	<0.001	4.52	1.54–17.13	0.012
- British Columbia	8.83	2.93–26.62		6.74	2.05–27.63	0.003
- Atlantic Canada ^g^	2.99	0.99–9.02		2.54	0.79–10.14	0.144
- Territories ^h^	(ref)			(ref)		
Geographic Location ^b^						
- Rural	1.4	0.95–2.06		1.06	0.63–1.76	0.815
- Remote	0.52	0.12–2.32	0.146	0.69	0.09–3.12	0.661
- Urban	(ref)			(ref)		
Income in year 2020 ^i^						
- <$20,000	4.34	2.41–7.80	<0.001	4.21	2.03–8.75	<0.001
- $20,000 to <$40,000	1.51	0.90–2.53		0.89	0.45–1.72	0.732
- $40,000 to <$60,000	1.51	0.98–2.32		1.17	0.67–2.00	0.583
- $60,000+	(ref)			(ref)		
Pre-pandemic mental health condition ^d^						
- Yes	10.93	7.08–16.88	<0.001	12.14	6.98– 21.66	<0.001
- No	(ref)			(ref)		
Presence of a chronic physical health condition ^d^						
- Yes	2.71	1.88–3.92	<0.001	2.22	1.34–3.67	0.002
- No	(ref)			(ref)		
Time since cancer diagnosis						
- <2 years	0.9	0.53–1.53	0.32	1.08	0.53–2.24	0.84
- 2 to <5 years	1.21	0.75–1.94		1.23	0.65–2.39	0.529
- ≥5 years	(ref)			(ref)		
Cancer type						
- Hematologic	0.95	0.61–1.48	0.828	0.83	0.43–1.54	0.569
- Non-hematologic ^j^	(ref)			(ref)		
Currently receiving cancer treatment ^c^						
- Yes	1.1	0.77–1.57	0.596	1.69	1.03–2.77	0.036
- No	(ref)			(ref)		

^a^*n* = 802 for univariable analysis; ^b^ *n* = 801 for univariable analysis; ^c^ *n* = 803 for univariable analysis; ^d^ *n* = 800 for univariable analysis, ^e^ Ontario, Quebec; ^f^ Alberta, Manitoba, Saskatchewan ^g^ Newfoundland and Labrador, Nova Scotia, New Brunswick, Prince Edward Island; ^h^ Yukon, Northwest Territories, Nunavut; ^i^ in Canadian Dollars; ^j^ Solid tumors and brain tumors.

**Table 3:** Themes and subthemes of optimization of cancer care during the COVID-19 pandemic (*n* = 235 excerpts).

Theme	Definition	Examples of Excerpts
Improving Healthcare Visits (37%)		
Increased ability to make appointments in advance (flexible and responsible scheduling)	Descriptions of ways care could best be arranged or facilitated by healthcare providers	“Make it easier to contact doctors/nurses through email/video calls”
More virtual appointments		“Easier access to doctors regarding appointments and bookings so that calls don’t go weeks with no response”
Providing care closer to home (decentralized care)		“Offers on-site therapy as well as telemedicine”
Enhancing Personalized Care (18%)		
Good communication from cancer organizations	Ideas about how their experience could be enhanced to support their wellbeing by healthcare teams	“Listen to us and do not ignore our symptoms”
Caring for and encouraging patients (compassion and validation)		“Continue to provide support services (as opposed to cancelling and closing most services during the pandemic)”
Access to physical rehabilitation		
Increased mental health supports		“Having access to support for mental health, coping, pain management”
Improving Prevention of COVID-19 (15%)		
Physical distancing	Suggestions on ways individuals and healthcare centers can prevent spreading or contracting COVID-19	“We should not go to places where people gather. If we can not go out, we should not go out” “Reduce the number of visits to the hospital”
Use of protective equipment and practices		“Wash your hands frequently and wear a mask”
Addressing Information Needs (13%)		
COVID-19-specific information	Statements describing optimal ways to receive information related to cancer care, self-care, or how the COVID-19 pandemic affects them specifically as a population	“Reach out to cancer patients directly with information on how the pandemic affects our particular cases”
In-person and online mediums		“Provide digital resources or connections at home (e.g., how to stay healthy and active)” “Cancer organizations provide better care by including information on the site”
Avoiding Diagnostic and Treatment Delays (11%)		
Delay in diagnosis Delay in treatment	Statements highlighting the importance of not delaying diagnostic tests, appointments, and treatment protocols	“COVID should have NO impact on testing and treatment, and care providers should be very open about why appointments are being rescheduled” “Do not delay follow-up appointments, treatments, or exams”

## Data Availability

The datasets generated during and/or analyzed during the current study are available from the corresponding author on reasonable request.

## Supplementary Materials

The following are available online at https://www.mdpi.com/article/10.3390/curroncol28040278/s1, Supplemental Appendix A: Informed consent and survey questions applicable to analysis of impact of COVID-19 pandemic on cancer care of AYAs with cancer; Table S1: Factors associated with a negative impact on cancer care among the participants receiving active cancer treatment (*n* = 265); Table S2: Factors associated with a negative impact on cancer care among the participants who had completed cancer treatment (*n* = 538).
