# Supplementary material for: A Cross-Sectional Survey Exploring the Impact of the COVID-19 Pandemic on the Cancer Care of Adolescents and Young Adults
Source: Curr Oncol. 2021 Aug 22;28(4):3201–13. doi: 10.3390/curroncol28040278 (PMC8395398; doi:10.3390/curroncol28040278)
Supplement: Supplementary file 1 [file curroncol-28-00278-s001.zip › curroncol-1324033-supplementary.pdf]

Article

A Cross-Sectional Survey Exploring the Impact of the COVID-19 Pandemic on the Cancer Care of Adolescents and Young Adults

Kaitlyn Howden [1,†], Camille Glidden [2,†], Razvan Romanescu [3], Andrew Hatala [4], Ian Scott [5], Julie Deleemans [6], Karine Chalifour [7], Geoff Eaton [7], Abha A. Gupta [8,9], James M. Bolton [2,10], Alyson L. Mahar [4,11], Sheila N. Garland [12] and Sapna Oberoi [1,13,14*]

Supplemental Appendix A: Informed consent and survey questions applicable to analysis of impact of COVID-19 pandemic on cancer care of AYAs with cancer

You are invited to take part in a research survey entitled:

"Impact of COVID-19 Pandemic on Adolescents and Young Adults with Cancer in Canada"

This form is a part of informed consent process. The information provided below should give you the basic idea of what this survey/study is about and what your participation will involve. It also describes your right to withdraw from the survey/study. In order to decide whether you wish to participate in this research study, you should understand enough about its risks and benefits and be able to make an informed decision. This is the informed consent process. Please read this information carefully and to understand the information given to you. Please contact Dr. Sapna Oberoi, Assistant professor, University of Manitoba at oberois@myumanitoba.ca or 204-787-4419 if you have any questions about the study or would like more information before you consent.

Purpose of this study:

The purpose of this study is to investigate the effect of COVID-19 on the cancer care, mental health and wellbeing of adolescents and young adults (AYAs) with cancer across Canada. We want to know how COVID-19 pandemic has affected your cancer care, physical health, mental health, relationships and behavior. We also want to know if the services provided to you by your cancer organization/hospital during this challenging pandemic were adequate or not.

Through this survey we also want to know how best the cancer organizations can support YAs with cancer during and after this pandemic. Data obtained from this study will be used to advocate for the needs of AYAs with cancer and inform cancer programming that better meets the needs of this population during and after this pandemic.

Are you Eligible to Participate?

To be eligible you must have been diagnosed with any type or stage of cancer after the age of 15 but before the age of 40. You must also currently be 18 year of age or older and living in Canada.

What you will do in this study:

In this study, you have been asked to complete an online survey. Participation will include answering questions about age, gender, family and academic/employment status, perceived risk of the COVID-19 virus on your health, cancer care and about your current state of physical health, psychological wellbeing and social relationships.

Length of time:

Completion of this online survey will take approximately 10-15 minutes.

Confidentiality & Anonymity:

This study has been reviewed and approved by the Research Ethics Board at the University of Manitoba and CancerCare Manitoba. Information obtained from this study will be anonymous. You will not be providing identifying information such as name, birth date, postal code, address or description of physical characteristics. Data collected will be stored in a password protected environment on the secure REDCap database at the University of Manitoba and at the CancerCare Manitoba. Only study team members will have access to the collected study data.

Reporting of Results:

Reports on the summary results of this survey will be published in research journals. The summary results will also be presented at various scientific conferences, meetings, cancer organizations and AYA support groups to use the information gained from this study to provide adequate resources and support to AYAs with cancer within Canada. As no identifying information is being collected in this survey, you will not be identified in any published data.

Sharing of Results with Participants:

The summary results of the survey will be made available on the CancerCare Manitoba's website https://www.cancercare.mb.ca/home/ and on YACC's website at www.youngadultcancer.ca.

Incentives

For your participation in the survey, you will be offered a 10-dollar Tim Horton's or Starbucks e- gift card. To get the e-gift card, simply click the link to the additional survey on the final page to fill out your email and preference of gift card (Tim Horton's or Starbucks). You must complete at least 80% of the survey to get this e-gift certificate. Entering your contact information on a separate page means we cannot link your identifying information back to your data and this will allow us to keep your responses anonymous. Those who qualify for an e-gift card will receive it once the survey has been closed to all participants.

Possible benefits:

Although you may not receive any immediate or direct benefits yourself, your participation will help us to better understand the specific issues that AYAs with cancer are facing during this pandemic. This in turn may be used to help organizations develop programs to address the unique issues faced by AYAs with cancer during this COVID-19 pandemic and even after the pandemic.

Possible risks:

It is possible that you may consider certain questions in this study to be sensitive. In this study, you will be asked to share information about your cancer experience during the pandemic and the effect this time has had on your psychological, social, and physical well- being. Should you feel distressed from participating in this research, or if you are concerned about your well-being, you are encouraged to contact your local Canadian Mental Health Association (CMHA). You can find your local CMHA at http://www.cmha.ca/get-involved/find- your-cmha/.

Withdrawal from the study:

Participation in this study is completely voluntary and if at any time you wish to discontinue the survey, you can simply click the EXIT button, which will be present on each page. If you feel uncomfortable providing any information, you can cancel your participation in this study with no negative consequences.

For questions about your rights as a research participant, you may contact The University of Manitoba, Bannatyne Campus Research Ethics Board Office at (204) 789-3389.

Consent:

By completing this survey, you agree that:

- You were diagnosed with any type of cancer after the age of 15 but before the age of 40.
- You are currently 18 years of age or older.
- You understand the purpose and intent of this research survey and what you will be doing.
- You have been advised that you may ask questions about this study at any time and receive answers prior to starting the survey
- You have not been unduly influenced by any study team member to participate in this research study.
- You are satisfied that any questions regarding this study you had have been addressed.
- You understand that participation is completely voluntary and you can withdraw at any point prior to or during completion of the survey without changing the quality of care that you receive.

By consenting to this online line survey, you have not waived any of the legal rights that you have as a participant in a research study.

Please retain a copy of this consent information for your records. You can download a copy of this form here.

Clicking 'NEXT' below and submitting this survey constitutes your informed consent and implies your agreement to the above statements.

Demographics and cancer treatment

1. What is your age in years? *Free text*
2. What is your gender
    a. Male
    b. Female
    c. Transgender
    d. Gender variant/Non-conforming
    e. I prefer not to answer
    f. Other (please specify)
3. How would you describe your relationship status?
    a. Single
    b. Common law/married
    c. Separated/divorced
    d. Widowed
    e. In a committed relationship

f. Other (please specify) *free text*

4. How would you describe your race/ethnicity? (please check all that apply)
    a. White
    b. Non-white
    c. I prefer not to answer
    d. Other, please specify
5. What province/territory do you live in?
    a. Alberta
    b. British Columbia
    c. Manitoba
    d. New Brunswick
    e. Newfoundland and Labrador
    f. Northwest Territories
    g. Nova Scotia
    h. Nunavut
    i. Ontario
    j. PEI
    k. Quebec
    l. Saskatchewan
    m. Yukon
6. How would you describe the location where you live?
    a. Urban
    b. Rural
    c. Remote
    d. I don't know
7. What is your current school/employment status? (please check all that apply)
    a. Part-time student
    b. Full-time student
    c. Working part-time
    d. Working full-time
    e. Unemployed
    f. Full-time homemaker or family caregiver
    g. Collecting disability or unemployment benefits
    h. Other (please specify) *free text*
8. Which of the following categories best describes your personal income in 2020?
    a. Less than $20,000
    b. $20,000 to less than $40,000
    c. $40,000 to less than $60,000
    d. $60,000 to less than $80,000
    f. $80,000 to less than $100,000
    g. $100,000 or more
    h. Not applicable as I am not working

9. When were you first diagnosed with cancer (year): *free text*
10. What type of cancer were you diagnosed with? (please select all that apply)
    a. Testicular cancer
    b. Breast cancer
    c. Thyroid cancer
    d. Leukemia
    e. Lymphoma
    f. Sarcoma
    g. Colon cancer
    h. Brain cancer
    i. Cervical cancer
    j. Melanoma
    k. Lung cancer
    l. Stomach cancer
    m. Liver cancer
    n. *Other, please specify*
11. What is your current cancer treatment status?
    a. Undergoing cancer treatment
    b. Completed treatment within the last 6 months
    c. Completed treatment within last 1 year
    d. Completed treatment within last 2 years
    e. Completed treatment within last 5 years
    f. Completed treatment within last 10 years
    g. Completed treatment within last 15 years
    h. Other, please specify
12. Did you have a pre-existing mental health condition before the pandemic?
    a. Yes
    b. No
    c. I prefer not to answer
13. If you have answered yes to question 17, then please specify the condition (please select all that apply)
    a. Anxiety disorders e.g., generalized anxiety disorder, panic disorders, phobias, post-traumatic stress disorders (PTSD), or obsessive-compulsive disorder (OCD)
    b. Mood disorders e.g., depression or bipolar disorders
    c. Other, please specify
    d. I prefer not to answer
14. Do you have any of following chronic health conditions (Please select all that apply)?
    a. lung disease
    b. heart disease
    c. hypertension (high blood pressure)
    d. diabetes

e. kidney disease
f. liver disease
g. stroke
h. Others, please specify
i. I don't have any chronic health condition

Effect of COVID-19 on cancer care

15. Did the COVID-19 pandemic have an impact on your cancer treatment or follow up appointments?
    a. Yes
    b. No
    c. Not yet, but I am concerned it will
16. If you answered "a" or "c" to question 15, please briefly provide details of the impact? (Please select all that apply)
    a. Change in the cancer treatment protocol
    b. Delay in cancer treatment
    c. Delay in the diagnostic tests
    d. Access to clinical trials
    e. Delay of the appointments
    f. Cancellation of the appointments
    g. Change of in-person appointment to phone appointments
    h. Change of in-person appointment to video appointments (Microsoft teams, webex or zoom)
    i. Change of in-person appointment to telehealth
    j. Delay or cancellation of other supports such as psychological, spiritual, physiotherapy, occupational therapy and vocational rehabilitation
    k. Other, please specify *free text*
17. How satisfied are you with the cancer care provided to you by your cancer organization during this pandemic?
    a. Very satisfied
    b. Satisfied
    c. Moderately satisfied
    d. Dissatisfied
    e. Very Dissatisfied
    f. Don't know
    g. I prefer not to answer
    h. Not applicable, as I don't have follow ups with cancer centre any more
18. How can cancer organizations provide optimal cancer care during this pandemic to adolescents and young adults with cancer? (free text)
19. What changes in cancer care delivery during the pandemic have had a **positive effect** on you? (please select all that apply)

a. Phone call visits
b. Telehealth visits
c. Video call visits e.g., Microsoft teams, Zoom etc.
d. Delivery of cancer care closer to home
e. Ability to communicate with health care providers by phone
f. Ability to communicate with health care providers by email
g. Others, please specify

Table S1: Factors associated with a negative impact on cancer care among the participants receiving active cancer treatment (n = 265).

	**Univariable Analysis (n = 265)**			**Multivariable Analysis (n = 222)**		
	**Odds Ratio**	**95% CI (lower-upper)**	***p*-Value**	**Adjusted Odds Ratio**	**95% CI (lower, upper)**	***p*-Value**
Age						
- > 25 years	0.92	0.56–1.52	0.756	0.66	0.21–2.22	0.480
- < 18–25 years	(ref)			(ref)		
Gender[a]						
- Woman	2.73	1.77–4.20	<0.001	0.76	0.28–1.94	0.567
- Man	(ref)			(ref)		
Ethnicity						
- Non-White	1.45	0.55–3.79	0.45	6.75	0.83–67.86	0.081
- White	(ref)			(ref)		
Province/Territory						
- Central Canada[e]	8.23	2.44–27.76		54.53	3.50–3200.10	0.019
- Prairies[f]	5.99	1.78–20.18	<0.001	16.64	1.13–867.45	0087
- British Columbia	8.07	2.20–29.54		31.09	1.70–1877.72	0.048
- Atlantic Canada[g]	2.72	0.75–9.88		10.57	0.61–589.87	0.165
- Territories[h]	(ref)			(ref)		
Geographic Location[b]						
- Rural/Remote	1.140	0.70–1.86	0.602	1.58	0.59–4.22	0.355
- Urban	(ref)			(ref)		
Income in year 2020[i]						
- <$20 000	3.89	1.86–8.11	0.002	5.91	1.52–23.35	0.01
- $20 000 to <$40 000	1.11	0.59–2.10		1.05	0.22–4.26	0.947
- $40 000 to <$60 000	1.52	0.92–2.51		0.71	0.16–2.51	0.610
- $60 000 +	(ref)			(ref)		
Pre-pandemic mental health condition[d]						
- Yes	9.05	5.51–14.86	<0.001	69.14	8.65–1850.99	<0.001
- No	(ref)			(ref)		
Presence of a chronic physical health condition[d]			<0.001			0.001
- Yes	2.15	1.38-3.36		6.88	2.18–22.14	
- No	(ref)			(ref)		
Time since cancer diagnosis						
- < 2 years	0.90	0.50–1.60	0.503	1.46	0.43–5.59	0.558
- 2 to < 5 years	0.70	0.36–1.35		0.67	0.17–2.75	0.564
- ≥ 5 years	(ref)			(ref)		
Cancer type						
- Hematologic	0.85	0.50–1.47	0.566	1.42	0.27–5.97	0.651
- Non-hematologic[j]	(ref)			(ref)		

[a] n = 802 for univariable analysis; [b] n = 801 for univariable analysis; [c] n = 803 for univariable analysis; [d] n = 800 for univariable analysis
[e] Ontario, Quebec; [f] Alberta, Manitoba, Saskatchewan [g] Newfoundland and Labrador, Nova Scotia, New Brunswick, Prince Edward Island; [h] Yukon, Northwest Territories, Nunavut; [i] in Canadian Dollars; [j] Solid tumors and brain tumors

Table S2. Factors associated with a negative impact on cancer care among the participants who had completed cancer treatment (*n*= 538).

	Univariable Analysis (*n* = 538)			Multivariable Analysis (*n* = 481)		
	**Odds Ratio**	**95% CI (lower-upper)**	***p*-Value**	**Adjusted Odds Ratio**	**95% CI (lower, upper)**	***p*-Value**
Age						
- > 25 years	0.46	0.24–0.88	0.018	0.72	0.38–1.40	0.317
- < 18–25 years	(ref)			(ref)		
Gender[a]						
- Woman	0.74	0.41–1.34	0.317	2.22	1.30–3.82	0.004
- Man	(ref)			(ref)		
Ethnicity						
- Non-White	2.86	0.74–11.0	0.112	1.64	0.47–5.38	0.424
- White	(ref)			(ref)		
Province/Territory						
- Central Canada[e]	5.44	1.11–26.63		7.89	2.35–37.36	0.003
- Prairies[f]	2.75	0.56–13.55	0.065	3.97	1.19–18.58	0.043
- British Columbia	3.75	0.64–22.1		7.09	1.81–36.73	0.009
- Atlantic Canada[g]	1.17	0.178–7.63		2.23	0.59–11.09	0.269
- Territories[h]	(ref)			(ref)		
Geographic Location[b]						
- Rural/Remote	1.57	0.86–2.90	0.140	0.82	0.42–1.53	0.538
- Urban	(ref)			(ref)		
Income in year 2020[i]						
- <$20 000	5.24	1.98–13.87	0.001	3.03	1.18–7.68	0.02
- $20 000 to <$40 000	3.03	1.24-7.42		0.61	0.26–1.36	0.245
- $40 000 to <$60 000	1.32	0.54–3.23		1.21	0.64–2.29	0.548
- $60 000 +	(ref)			(ref)		
Pre-pandemic mental health condition[d]						
- Yes	38.4	10.9–134.74	<0.001	10.41	5.66–19.72	<0.001
- No	(ref)			(ref)		
Pre-pandemic chronic physical health condition[d]						
- Yes	5.0	2.53–9.87	<0.001	1.40	0.75–2.57	0.281
- No	(ref)			(ref)		
Time since cancer diagnosis						
- < 2 years	2.18	0.96–4.95	0.116	0.94	0.43–2.15	0.888
- 2 to < 5 years	1.36	0.57–3.25		1.11	0.44–2.84	0.824
- ≥ 5 years	(ref)			(ref)		
Cancer type						
- Hematologic	1.23	0.56–2.69	0.609	0.81	0.39–1.00	0.547
- Non-hematologic[j]	(ref)			(ref)		

[a] *n* = 802 for univariable analysis; [b] *n* = 801 for univariable analysis; [c] *n* = 803 for univariable analysis; [d] *n* = 800 for univariable analysis
[e] Ontario, Quebec; [f] Alberta, Manitoba, Saskatchewan [g] Newfoundland and Labrador, Nova Scotia, New Brunswick, Prince Edward Island; [h] Yukon, Northwest Territories, Nunavut; [i] in Canadian Dollars; [j] Solid tumors and brain tumors
